# A plant cell‐based platform for the expression of complex proteins with fucose‐reduced sialylated N‐glycans

**DOI:** 10.1111/pbi.70044

**Published:** 2025-04-10

**Authors:** Saeideh Dianatkhah, Benjamin Kogelmann, Stanislav Melnik, Florian Eminger, Somanath Kallolimath, Lin Sun, Delia Sumesgutner, Michael W. Traxlmayr, Markus Sack, Eva Stoger, Herta Steinkellner

**Affiliations:** ^1^ Department of Applied Genetics and Cell Biology BOKU University Vienna Austria; ^2^ acib – Austrian Centre of Industrial Biotechnology Vienna Austria; ^3^ Department of Chemistry, Institute of Biochemistry BOKU University Vienna Austria; ^4^ CD Laboratory for Next Generation CAR T Cells Vienna Austria; ^5^ Pro‐SPR GmbH Alsdorf Germany; ^6^ Present address: Institute of Biotechnology Shiraz University Shiraz Iran

**Keywords:** plant cell packs, protein sialylation, *Nicotiana benthamiana*, recombinant glycoproteins

Sialylated N‐glycans are widely distributed in vertebrates and represent the dominant glycoform of many human plasma proteins (Miura *et al*., 2015). Although knowledge of the diverse effects of this glycan formation is rapidly increasing, full understanding of its biological significance remains elusive (Lewis *et al*., 2022). A major reason for this is the difficulty in controlling sialylation in production processes.

Plants are considered as an effective platform for the production of recombinant proteins used in basic research or for various applications (Eidenberger *et al*., 2023). The platform has recently been extended by so‐called plant cell packs (PCPs), three‐dimensional, porous plant cell aggregates derived from plant suspension cells. The approach enables high‐throughput transient expression of foreign genes and upscaling for subsequent protein purification and characterization (Rademacher *et al*., 2019) and (WO2013113504).

An important advantage of plant‐based expression is the synthesis of N‐glycans similar to mammalian cells. Usually, secreted plant glycoproteins are decorated with GlcNAc‐terminating complex N‐glycans carrying a plant‐specific core xylose and α1,3‐fucose, so‐called GnGnXF structures. Extensive engineering in *N. benthamiana*, that is, the inactivation of genes responsible for the addition of plant‐specific core xylose and fucose, in combination with the overexpression of six foreign genes involved in the human sialylation pathway, resulted in the generation of a plant line (ΔXTFT^Sia^) that synthesizes sialylated N‐glycans (Eidenberger *et al*., 2022; Kallolimath *et al*., 2016). One shortcoming of the ΔXTFT^Sia^ line is the lower seed production, which makes maintenance and widespread use difficult.

Here we used hypocotyl of ΔXTFT^Sia^ plants as starting material for callus induction, applying a similar method as described recently (Sukenik *et al*., 2018) (Figure S1a, ‘Materials and methods’ section). After tissue dedifferentiation, calli were maintained on semi‐solid media by monthly subculturing. Portions of independent calli, PCR‐screened for the presence of one of the six foreign glycosylation genes for sialylation (Figures S1a and S1b), were used to initiate suspension cultures, which were maintained for several passages to select for rapid growth (Figure S1a). For the generation of PCPs (Rademacher *et al*., 2019), cells were separated from excess cultivation medium by slow centrifugation in ultrafiltration spin‐columns The resulting semi‐dry porous cell aggregates (called plant cell packs, PCP^Sia^; Figure S1a) were monitored for expression of recombinant fluorescent protein. PCP^Sia^ were incubated with *Rhizobium radiobacter* (formerly *Agrobacterium tumefaciens*) suspension cultures carrying a DNA construct for the expression of monomeric red fluorescent protein (mRFP; Schoberer *et al*., 2019). Macroscopic inspection revealed specific fluorescent signals which were absent in cells that were infiltrated with agrobacteria carrying a non‐related gene (Figure S2). The results demonstrate the expression of a functionally active foreign gene in the PCP^Sia^ under the chosen settings.

Next, we aimed at the expression of a more complex protein of therapeutic relevance and chose cetuximab (Cx), a therapeutic monoclonal IgG1 antibody. Functionally active IgG1 requires simultaneous expression of two genes coding for heavy (HC) and light (LC) chains, as well as extensive protein folding and hetero‐dimeric assembly. Importantly, the Cx‐HC carries an additional glycosite (GS) in the Fab‐domain next to the conserved Fc‐GS. Fab GSs are usually more exposed to N‐glycan processing, which provides a benefit in monitoring elongations towards sialylation (Castilho *et al*., 2015). An up‐scaled format of PCPs called plant cell cookie (PCC^Sia^) with an increased surface‐to‐volume ratio to promote oxygen availability was applied (Figure S1a). Different intercellular liquid content of the PCC^Sia^ was tested, where it turned out that ‘low’ (i.e. complete removal of liquid) is advantageous because higher liquid content led to excessive unwanted browning of the PCC^Sia^ (‘medium’ and ‘high’) (Figure S3).

A potent transient expression vectors carrying respective Cx genes (pTra‐Cx) was delivered to PCC^Sia^ (Eidenberger *et al*., 2022). Western blotting of total soluble leaf proteins (TSP) exhibited specific HC and LC signals, which were especially high in one of the tested PCC^Sia^ samples, assigned as #3 (Figure 1a). These cells were subsequently used for Cx purification by protein A immunoaffinity and monitored by SDS‐PAA gel electrophoresis (Figure 1b). HC glycosylation profiles were determined by liquid chromatography‐electrospray ionization‐tandem mass spectrometry LC‐ESI‐MS (Figures 1c and S5 and S4, S6): Fc‐GS carried ~40% complex structures of which 7% were sialylated. No fucose‐ or xylose‐carrying structures were detected. In addition, 25% mannosidic and 31% single GlcNAc structures were present. Fab‐GS carried 65% complex structures, of which 60% were sialylated. Of note, about 30% of all complex N‐glycans were fucosylated. In addition, 15% mannosidic and 17% single GlcNAc structures were detected. Single GlcNAcs were a surprise and were not detected on Cx produced in plant leaves, neither in ΔXTFT nor in ΔXTFT^Sia^ – the PCC^Sia^ parental line (Castilho *et al*., 2015; Eidenberger *et al*., 2023). While Fab‐GS was fully occupied, 10–15% of Fc‐GS was non‐glycosylated. In addition, Cx samples derived from other PCCs were purified, and some of them lacked sialylated N‐glycans (Figure S5).

**Figure 1:**
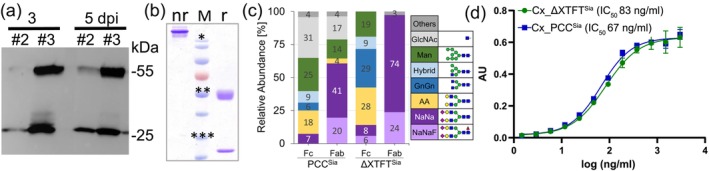
Monitoring recombinant protein expression. (a) Western blot analysis of total soluble proteins (TSP) extracted from Cx‐transformed PCP harvested 3 and 5 days post infiltration (dpi). Cells were derived from two independent calli (No. 2 and 3 on Figure S1a). In each lane approx. 50 μg TSP was loaded, (b) Coomassie blue‐stained SDS‐PAGE of purified Cx (derived from the #3) under reducing (r) and non‐reducing (nr) condition. Approx. 4 μg protein was loaded; M = Marker: * 120, ** 55, *** 35 kDa. (c) LC‐ESI‐MS/MS N‐glycosylation analysis results of Cx‐HC shown in (b) derived from ΔXTFT^Sia^ cookies #3 (PCC^Sia^) (Fig S1a) and from plant leaves. Bars represent the relative abundance (%) of glycoforms (for details see Figures S5 and S6). Nomenclature according to (Altmann *et al*., 2024). (d) hEGFR antigen binding of Cx produced in PCC^Sia^ and ΔXTFT^Sia^.

While the overall glycan composition was similar to Cx from PCC^Sia^ #3, complex glycans terminated with either GlcNAc or, to a lesser extent, with galactose (Figure S5). Apparently, during the laborious transition process, that lasted about 12 months (from callus induction to the purified mAb) one or more of the foreign genes were lost or became inactive. For comparison, the glycosylation profile of Cx expressed in the parental line ΔXTFT^Sia^ was evaluated (Figures 1c and S5 and S6). Compared to PCC‐Sia #3 a similar glycan composition was observed with three striking differences: the degree of sialylation was higher, with up to 98% (at the Fab‐GS), no single GlcNAc was present and the fraction of non‐occupied Fc‐GS was significantly higher (up to 35%). Such unexpected modifications are most probably associated with genetic modifications that occurred along the transition process, a phenomenon recognized in a previous study (Tanurdzic *et al*., 2008). Current outcomes may reflect alterations in the expression of oligosaccharyltransferase complex responsible for the en bloc transfer of the conserved oligosaccharide Glc3Man9GlcNAc2 to nascent polypeptide chains (Beihammer *et al*., 2023; Jeong *et al*., 2018) or de‐glycosylation enzymes like ENGases (Rademacher *et al.*, 2008; Vuylsteker *et al*., 2000). Also, high recombinant production might saturate the system in ER and subsequently alter post‐translational modifications. Finally, an ELISA‐based antigen binding assay revealed similar binding activities of PCC^Sia^‐ and ΔXTFT^Sia^‐ derived Cx, demonstrating full functional integrity of mAb produced in PCC^Sia^ (Figure 1d).

Collectively, here we show the establishment of a plant cell line that enables the transient expression of human multi‐component proteins with reduced fucosylation and targeted sialylation – one of the most complex human N‐glycan modifications. To the best of the author's knowledge, this is unique among the established production systems, since they either lack the entire N‐glycosylation machinery (i.e., microbes) or carry an exuberant endogenous glycosylation repertoire (mammalian cells). Notably, IgG‐Fab represents an exposed GS as it is mostly the case for therapeutically‐relevant proteins, like EPO, alpha‐1‐antitrypsin, or Fc‐fusion‐based decoy receptors (Keshvari *et al*., 2024). Among the many GSs analysed on different proteins, IgG‐Fc provides an exception due to its structural peculiarities (Castilho *et al*., 2015). The presence of the core α1,3‐linked fucose is absolutely required for efficient sialylation. In case where high Fc sialylation is required, a *FucT* gene can be co‐delivered in the agrobacterial infiltration mix (Castilho *et al*., 2015; Eidenberger *et al*., 2022).

With the present work we augment cell‐based expression platforms with a rapid screening and scalable technology for the expression of glycoproteins.

## Author contributions

Saeideh Dianatkhah: Data curation, formal analysis, investigation, visualization, validation, writing – original draft; Benjamin Kogelmann: Data curation, formal analysis, investigation, validation, visualization, writing – original draft, writing – review and editing; Stanislav Melnik: Data curation, formal analysis, investigation, methodology, validation, visualization, writing – original draft, writing – review and editing; Florian Eminger: Formal analysis, data curation, investigation, writing – original draft; Somanath Kallolimath: Data curation, formal analysis, writing – review and editing; Lin Sun: Investigation, formal analysis, writing – review and editing; Delia Sumesgutner: formal analysis, generated materials; Michael W. Traxlmayr: providing material, writing – original draft; Markus Sack: Methodology, writing – review and editing; Eva Stoger: Conceptualization, supervision, writing – review and editing; Herta Steinkellner: Conceptualization, project administration, funding acquisition, supervision, writing – original draft, writing – review and editing.

## Conflict of interest

The authors declare that the research was conducted in the absence of any commercial or financial relationships that could be construed as a potential conflict of interest. MWT receives funding from Miltenyi Biotec, GER.

## Supporting information


**Table S1** Media for tissue culture.
**Figure S1** Schematic illustration of plant cell pack generation (a) Callus generation from aseptically grown hypocotyl‐derived from ΔXTFT_Sia_ plants with subsequent transfer to suspension cell culture of dedifferentiated explants, workflow of transfer to suspension culture and casting of a plant cell pack (PCP, see also Rademacher et al., 2019) or plant cell cookies (PCC). (b) PCR results of different ΔXTFT^Sia^‐derived calli detecting GNE gene (amplicon size 580 bp) and the house keeping gene catalase (amplicon size 500 bp). Expected position of the specific amplicons are indicated with a black triangle; M1: Marker – 1 kb DNA ladder (NEB); M2: Marker – 100 bp DNA ladder (NEB).
**Figure S2** mRFP expression (bacterial OD_600_ of 0.5) in PCP^Sia^ monitored 9 dpi by macroscopic fluorescence using a red light/green filter combination (see material and methods). ‐ ctr: negative control, refers to infiltration with a construct carrying an irrelevant protein.
**Figure S3** Intercellular liquid content of PCC_Sia_. Low: complete removal of excess liquid without humidity chamber; medium: partial removal of excess liquid with humidity chamber; High: adding additional drops of water on the filter paper and humidity chamber.
**Figure S4** Peptide mass‐fingerprint analysis and sequence coverage of PCC^Sia^ derived cetuximab heavy chain (A) and light chain (B). Sequence covered is highlighted in gray boxes in bold and the observed individual peptides are represented as blue lines underlying their respective matching sequence; carbamidomethylated cysteines (+57.02 Da) and oxidized methionines (+15.99 Da) are highlighted in red and orange, respectively.
**Figure S5** LC‐ESI‐MS based quantified glycoforms of purified cetuximab (Cx) Fc and Fab glycosite. Glycan nomenclature according to Altmann *et al*. (2024).
**Figure S6** Examples for LC‐ESI‐MS spectra of Cx‐Fcs and Fab (deconvoluted form) expressed in (a) PCC^Sia^ and in (b) ΔXTFT^Sia^. Fc glycopeptide EEQYNSTYR (1189.5120 Da) with glycosite at N297 and Fab glycopeptide MNSLQSNDTAIYYCAR (1906.8422). Glycan nomenclature according to Altmann *et al*. (2024).

## Data Availability

Data sharing not applicable to this article as no datasets were generated or analysed during the current study.
