# Supplementary material for: A plant cell‐based platform for the expression of complex proteins with fucose‐reduced sialylated N‐glycans
Source: Plant Biotechnol J. 2025 Apr 10;24(1):93–5. doi: 10.1111/pbi.70044 (PMC12854884; doi:10.1111/pbi.70044)
Supplement: Supplementary file 1 — Table S1 Media for tissue culture. Figure S1 Schematic illustration of plant cell pack generation (a) Callus generation from aseptically grown hypocotyl‐derived from ΔXTFTSia plants with subsequent transfer to suspension cell culture of dedifferentiated explants, workflow of transfer to suspension culture and casting of a plant cell pack (PCP, see also Rademacher et al., 2019) or plant cell cookies (PCC). (b) PCR results of different ΔXTFTSia‐derived calli detecting GNE gene (amplicon size 580 bp) and the house keeping gene catalase (amplicon size 500 bp). Expected position of the specific amplicons are indicated with a black triangle; M1: Marker – 1 kb DNA ladder (NEB); M2: Marker – 100 bp DNA ladder (NEB). Figure S2 mRFP expression (bacterial OD600 of 0.5) in PCPSia monitored 9 dpi by macroscopic fluorescence using a red light/green filter combination (see material and methods). ‐ ctr: negative control, refers to infiltration with a construct carrying an irrelevant protein. Figure S3 Intercellular liquid content of PCCSia. Low: complete removal of excess liquid without humidity chamber; medium: partial removal of excess liquid with humidity chamber; High: adding additional drops of water on the filter paper and humidity chamber. Figure S4 Peptide mass‐fingerprint analysis and sequence coverage of PCCSia derived cetuximab heavy chain (A) and light chain (B). Sequence covered is highlighted in gray boxes in bold and the observed individual peptides are represented as blue lines underlying their respective matching sequence; carbamidomethylated cysteines (+57.02 Da) and oxidized methionines (+15.99 Da) are highlighted in red and orange, respectively. Figure S5 LC‐ESI‐MS based quantified glycoforms of purified cetuximab (Cx) Fc and Fab glycosite. Glycan nomenclature according to Altmann et al. (2024). Figure S6 Examples for LC‐ESI‐MS spectra of Cx‐Fcs and Fab (deconvoluted form) expressed in (a) PCCSia and in (b) ΔXTFTSia. Fc glycopeptide EEQYNSTYR (1189.5120 Da) with [file PBI-24-93-s001.docx]

**Supporting information for**

A plant cell-based platform for the expression of complex proteins with fucose-reduced sialylated N-glycans

Saeideh Dianatkhah^a,b^, Benjamin Kogelmann^a,c^, Stanislav Melnik^a,c^, Florian Eminger^a^, Somanath Kallolimath^a^, Lin Sun^a^, Delia Sumesgutner ^d,e^, Michael W. Traxlmayr^d,e^, Markus Sack^f^, Eva Stoger^a^ and Herta Steinkellner^a,*^

^a^ Department of Applied Genetics and Cell Biology, BOKU University, Vienna, Austria

^b^ Current address Institute of Biotechnology, Shiraz University, 71441-65186 Shiraz, Iran

^c^ acib - Austrian Centre of Industrial Biotechnology, Muthgasse 18, 1190 Vienna, Austria.

^d^ Department of Chemistry, Institute of Biochemistry, BOKU University, Vienna, Austria

^e^ CD Laboratory for Next Generation CAR T Cells, Vienna, Austria.

^f^ Pro-SPR GmbH, Goethestrasse 34, 52477 Alsdorf, Germany.

*Correspondence:

Herta Steinkellner

Department of Applied Genetics and Cell Biology, University of Natural Resources and Life Sciences, Vienna, Muthgasse 18, 1190 Vienna, Austria

Tel: +43-1-47654-94370,

Fax: +43-1-47654-94009,

**Email:** [herta.steinkellner](mailto:herta.steinkellner)@boku.ac.at

**Material and Methods**

**Callus generation of *N. benthamiana* mutant ΔXTFT^Sia^**

Seeds of the *N. benthamiana* ΔXTFT^Sia^ (Kallolimath et al., 2016) were sterilized by incubation in 70% ethanol for 30 seconds and 5% sodium hypochlorite containing 0.5% Tween-20 for 20 minutes on a rotating wheel. Then the seeds were washed five times with sterile water and were germinated on ½ MS plates. The epicotyl of 7-day old seedlings was cut with a sharp and sterile razorblade and placed on callus generation plates (see Table S1). After 3 weeks, hypocotyl with induced calli were transferred to callus maintenance plates (0.2 mg/L 2,4-dichlorophenoxyacetic acid Table S1). For maintenance, fresh and light-colored parts of the calli were transferred to new plates every month (Table S1).

**PCR screening**

DNA from calli and the parental line ΔXTFT^Sia^ were extracted as described (Kogelmann et al., 2024), and used for the detection of UDP-N-acetylglucosamine-2-epimerase/N-acetylmannosamine kinase (GNE) gene (GNE_F: ggatgccctgatctcgttta and GNE_R: aacgatttcacccttcatgc). *N. benthamiana* catalase gene (NbCat_F: GAGAGGTAATTTGCCCCATC and NbCat_R: ATTTGGGTTCTGCTTTGCC) was used as positive control.

**Cell suspension culture of ΔXTFT^Sia^**

To initiate cell suspension cultures, portions of calli were used to inoculate 50 ml suspension culture medium (Table S1) in 500 ml flasks. The cells were grown on a benchtop shaker in slow mode (120 rpm) at 25-28°C in the dark. Several cultures were grown until they reached approx. 50% cell density and then taken through several passages with weekly transfer by subculturing 30% of the previous culture by volume.

To test cell viability, 200 µL of suspension culture cells were treated with 2 µL of Evans blue staining solution (1% w/v Evans blue dye, 0.1 M CaCl_2_ pH 5.6 HCl) for 10 minutes shaking at 25°C. Then the cells were washed three times with sterile deionized water (centrifugation at low speed, 600 x g) and cell staining was examined under the microscope. Control cells were treated with 200 µl 70% ethanol for 3 hours.

**Preparation of plant cell packs (PCP) and plant cell cookies (PCC)**

First, plant cells were grown until a sufficient cell density of ~50 to 70 % packed cell volume was reached (~5 to 10 days, shaking at 25-28°C in the dark).

Plant cell packs (PCPs) were prepared similarly as described (Rademacher et al., 2019). In brief, the cells were resuspended by slow shaking and transferred to ultrafiltration spin-columns with a 0.45 µm cut-off (Merck Milipore Cat Nr 20-218) using serological pipettes. The excess liquid was removed by centrifugation at 600 x g for 5 minutes until the PCP was dry. The procedure was repeated until a final PCP cell volume of ~400 µl was reached.

For the preparation of plant cell cookies (PCC; i.e. PCP with increased mass), the suspension cells were filtrated on a Büchner funnel/filter paper (Whatman)/vacuum pump to completely remove the media and then subjected to co-cultivation with Rhizobium radiobacter (syn. Agrobacterium fabrum, formerly Agrobacterium tumefaciens; strain GV3101(pMP90)). Cells were stripped of liquid medium by collecting them on a filter paper placed into a Büchner funnel. The collected cells were re-suspended in co-cultivation buffer containing agrobacteria and incubated for 30-60 minutes. The cells were then collected and the paper filter with compacted layer of cells on top of it was placed in a Petri dish (Figure 1b).

**Expression of recombinant proteins**

The DNA expression constructs carrying mRFP (Schoberer et al., 2019) or Cetuximab (Cx) in a pTra backbone (Eidenberger et al., 2022) were delivered to plant cells via agroinfiltration. For this o/n cultures were pelleted (3000 x *g*, 5 minutes) and resuspended in the original culture volume of MS medium (1x MS salts, 3% w/w sucrose, pH 5.6) supplemented with 200 µM acetosyringone. After 30 minutes of incubation (in the dark, slow shaking) the OD_600_ was adjusted to 0.2 or 0.5. Subsequently the PCPs were submerged in bacterial solution and incubated for 30 minutes at room temperature (RT). Then the liquid was removed by centrifugation (600 x g, 5 minutes) and the column was sealed and incubated for 4 to 10 days at 20-22°C (RT) in a humidity chamber (perlite mixed with water). The mRFP expression was monitored visually by using a green light source (560 nm CWL, 13 nm FWHM) for excitation and a red long pass filter (605 nm cut-on) to capture the emitted signal.

For agroinfiltration in PCCs, o/n agrobacterium cultures were used to inoculate (1% v/v) LB media containing antibiotics, 20 mM MES and 50 μM acetosyringone and incubated o/n (29°C, shaking at 180 rpm). Then the culture was centrifuged at 3000 × g for 10 minutes and pellets resuspended to a the OD_600_ of 0.2 in sterile distilled MS medium containing 10 mM MgCl_2_, 20 mM MES and 150 μM acetosyringone. After a 3-hour incubation period at room temperature (22°C) 100 mL of the resuspended agrobacterium solution was added to the collected plant cells to reach ratio of wet cell mass to volume of agrobacterium suspension of ~1:3. The plant cell and agrobacterium suspension was then placed for 5 minutes on a shaker with 140 rpm in the dark followed by an incubation at 22°C for 30 to 60 minutes without shaking in the dark. Then ~30 to 40 mL of the suspension was filtrated via a Büchner funnel to remove excess liquid. For “low” intercellular liquid content the media was completely removed. The plant cell cookies including the filter paper were transferred in a petri dish. For “medium” and “high” (addition of liquid drops) intercellular liquid content the petri dish was stored in a humidity chamber (box with wet perlite) for 1-2 days in 20-22°C (RT). Subsequently, the plates were sealed loosely with parafilm, tightly closed after 1-2 days if the humidity decreased and transferred to 25°C for 4 to 10 days until samples for protein extraction were taken.

**Protein extraction, antibody purification**

300 mg of cells from PCC^Sia^ were snap-frozen in liquid nitrogen and crushed to fine powder and total soluble proteins (TSP) extracted incubated for 15 minutes on ice in 600 µL extraction buffer (0.1 M Tris, 0.5 M NaCl, 1 mM EDTA; 40 mM ascorbic acid; pH 7.4). After two centrifugation steps (13000 x g at 10 minutes) 300 µl of the supernatant was mixed with 1200 µl of acetone (1:4 ratio) and incubated at -20°C o/n. The extract was centrifuged at 10 minutes and the pellet resuspended in 30 µL 1x PBS (137 mM NaCl, 2.7 mM KCl, 10 mM Na_2_HPO_4_, 1.8 mM KH_2_PO_4_; pH 7.4) containing 1x reducing Lämmli buffer and boiled at 95°C for 5 minutes and 20 µL were used for SDS-PAGE and Western blotting, using anti-hIgG-HRPO (Anti-Human IgG (H+L), Promega Cat. Nr: W4031) and ECL substrate for detection. After verification of the expression by western blotting, the remaining PCC^Sia^ was homogenized by mortar and pestle, dissolved in TSP extraction buffer (~30-50 ml) and used to purify recombinant Cx by protein A affinity chromatography as recently described (Kogelmann et al., 2024).

**N-Glycan Analyses**

The N-glycosylation profiles of the purified Cx were determined by mass spectrometry (MS) as described previously (Kallolimath 2021, Sun 2021). Briefly, HC bands, excised from an SDS-PAGE gel, or fully assembled Cx in solution, were digested with trypsin and analyzed with an LC-ESI-MS system (Thermo Orbitrap Exploris 480). The glycopeptides were identified as sets of peaks consisting of the peptide moiety and the attached N-glycan varying in the number of HexNAc units, hexose, deoxyhexose, pentose and sialic acid residues. Manual glycopeptide searches were performed using FreeStyle 1.8 (Thermo), deconvolution was done using the extract function. The peak heights roughly reflect the molar ratios of the glycoforms and the unglycosylated peptide respectively. Nomenclature used was according to (Altmann et al., 2024).

**ELISA**

Binding of Cx to human EGFR was performed as previously described with modifications (Patel et al., 2007; Sun et al., 2021). 96-well plates (Thermo fisher maxisorp, catlog No: M9410-1CS) were coated using 50 μL/well of 2.0 μg/mL of hEGFR-Fc (expressed and purified as described in (Dobersberger et al., 2024) dissolved in PBS (pH 7.4) overnight at 4°C. The plate was washed four times using the washing buffer solution (PBS pH 7.4 containing 0.05% (v/v) Tween^®^ 20). The plate was then treated with 100 μL of the blocking buffer (PBS pH 7.4 containing 3% (w/v) skimmed milk) per well for 1.5 hours at RT. Following four times wash with PBST (PBS with 0.05% Tween) as explained above and filled with 50 μL of plant produced Cx diluted with blocking buffer in two-fold ranging from 3000 ng/mL to 1.5 ng/mL. After incubating for 2 hours at RT, the plate was washed four times with PBST and incubated with 50 μL/well of peroxidase-labeled anti-human kappa light chain (Sigma A7164) diluted in blocking buffer (1:20,000) for 1 hour at RT. After washing four times with PBST, 50 μL of TMB (Thermo Fisher, J61325.AU) was added to each well and incubated in darkness at RT. The reaction was stopped by adding 50 μL of 2 M sulfuric acid to each well. The absorbance was recorded at wavelengths of 450 nm and 620 nm using a Tecan Spark® spectrophotometer. IC_50_ values were calculated by non-linear regression of the blank-corrected data points based on a four-parametric log model with GraphPad Prism (version 9).

**Supporting tables**

**Table S1 Media for tissue culture**

| **Media** | **Components** |
| --- | --- |
| Callus generation medium | 1x MS Basal Salts, 30 g/l sucrose, 1x MS Vitamin Mixture (Duchefa M0409); Phytagel 2.5 g/l, pH 5.6 KOH; 1x MS Vitamin Mixture (Duchefa M0409), +1 mg/l supplemented Thiamine hydrochloride; 0.204 g/l potassium phosphate monobasic KH_2_PO_4_ buffer pH 5.7; 0.4 mg/l 2,4-D (Dichlorophenoxyacetic acid); 0.1 mg/l Kinetin |
| Callus maintenance medium | 1x MS Basal Salts, 30 g/l sucrose, 1x MS Vitamin Mixture (Duchefa M0409); Phytagel 2.5 g/L, pH 5.6 KOH; 1x MS Vitamin Mixture (Duchefa M0409), 0.204 g/L potassium phosphate monobasic KH_2_PO_4_ buffer pH 5.7; 0.2 mg/l 2,4-D (Dichlorophenoxyacetic acid); 0.1 mg/l Kinetin; supplemented with 1 mg/l Thiamine hydrochloride |
| Nb suspension culture medium | 1x MS Basal Salts, 30 g/l sucrose, 1x MS Vitamin Mixture (Duchefa M0409); pH 5.6 KOH; 1x Gamborg B5 Vitamins (Duchefa G0415); 0.204 g/l KH_2_PO_4_ pH 5.7; 2 mg/l 2,4-D (Dichlorophenoxyacetic acid); 0.1 mg/l Kinetin; supplemented with 9 mg/l nicotinic acid; 4 mg/l pyridoxine hydrochloride |

**Supporting figures**

| **(a)** |
| --- |
| 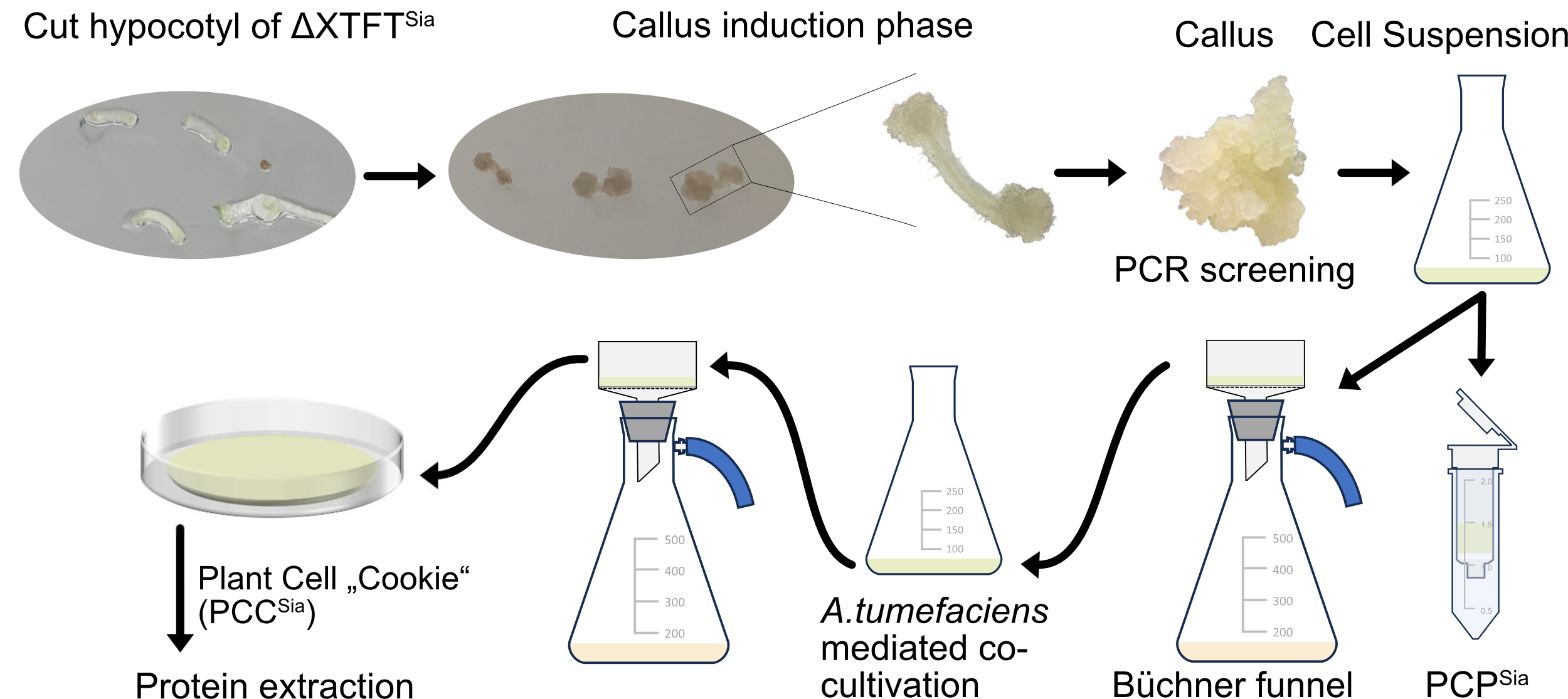 |
| **(b)** |
| **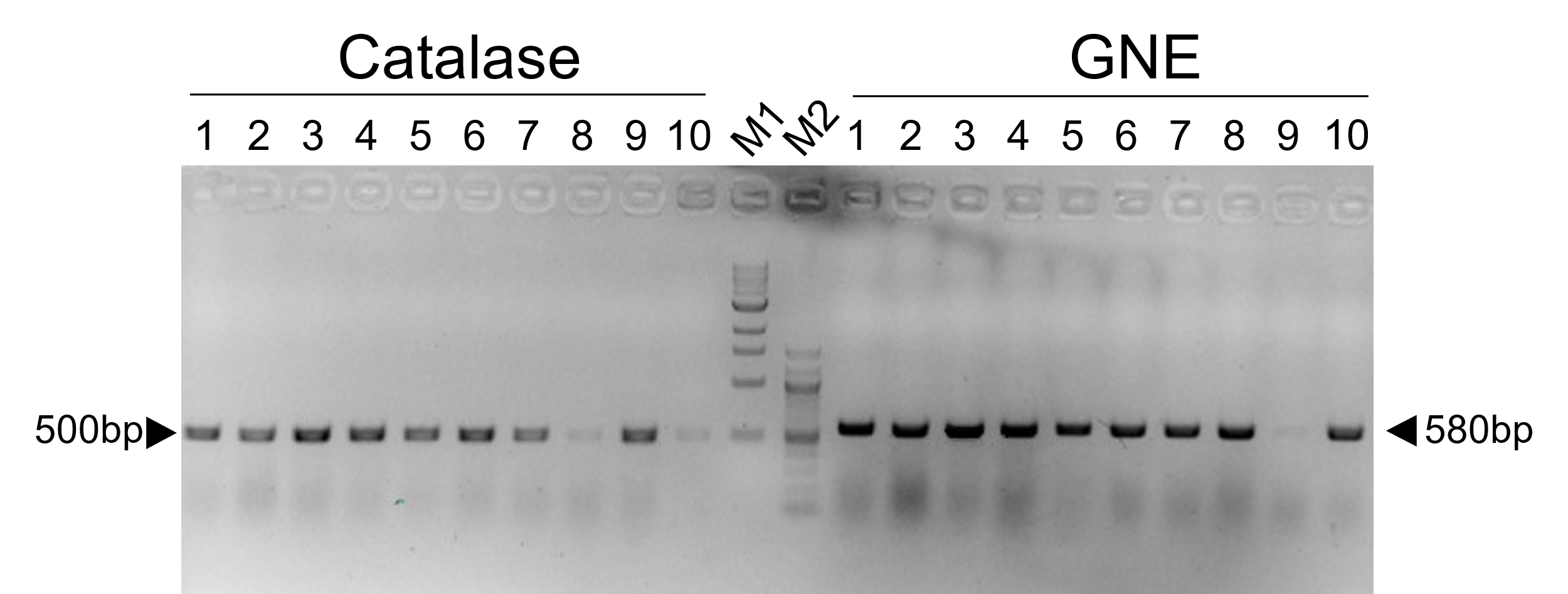** |

**Figure S1 Schematic illustration of plant cell pack generation .** (a) Callus generation from aseptically grown hypocotyl-derived from ΔXTFT^Sia^ plants with subsequent transfer to suspension cell culture of dedifferentiated explants, workflow of transfer to suspension culture and casting of a plant cell pack (PCP, see also Rademacher et al., 2019) or plant cell cookies (PCC). (b) PCR results of different ΔXTFT^Sia^-derived calli detecting GNE gene (amplicon size 580bp) and the house keeping gene catalase (amplicon size 500bp). Expected position of the specific amplicons are indicated with a black triangle; M1: Marker – 1kb DNA ladder (NEB); M2: Marker – 100bp DNA ladder (NEB).

| **-ctrl** | **mRFP** |  |
| --- | --- | --- |
| **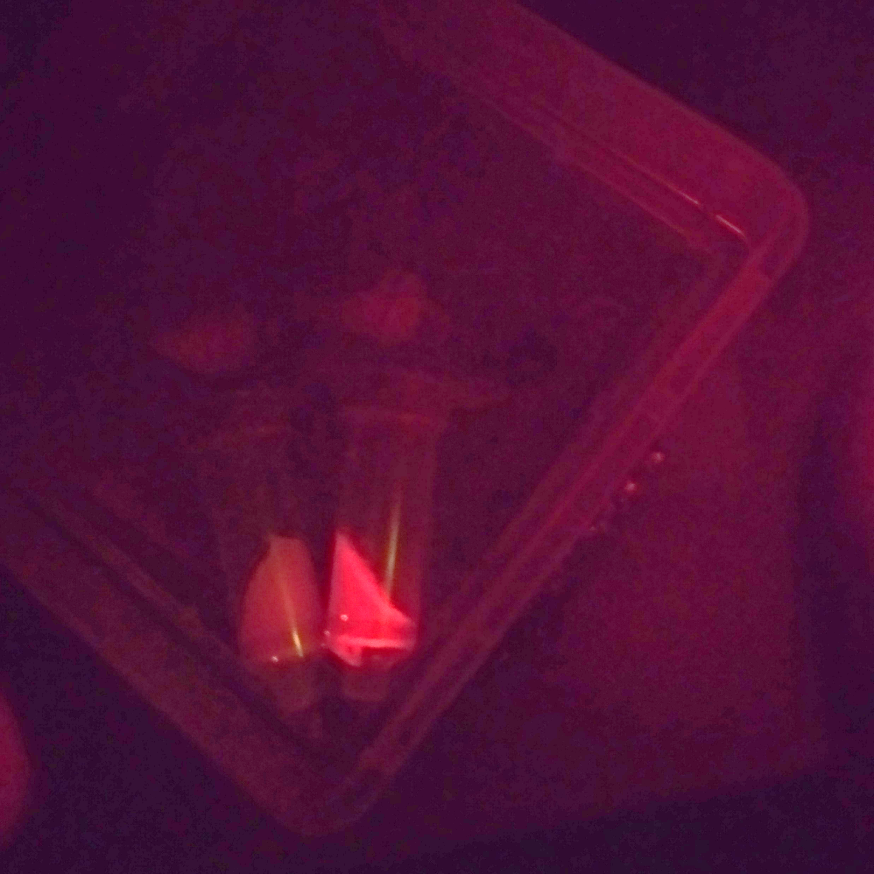** | | |

**Figure S2 mRFP expression** (bacterial OD_600_ of 0.5) in PCP^Sia^ monitored 9 dpi by macroscopic fluorescence using a red light/green filter combination (see material and methods). – ctr: negative control, refers to infiltration with a construct carrying an irrelevant protein.

|  | low | medium | high |
| --- | --- | --- | --- |
|  | 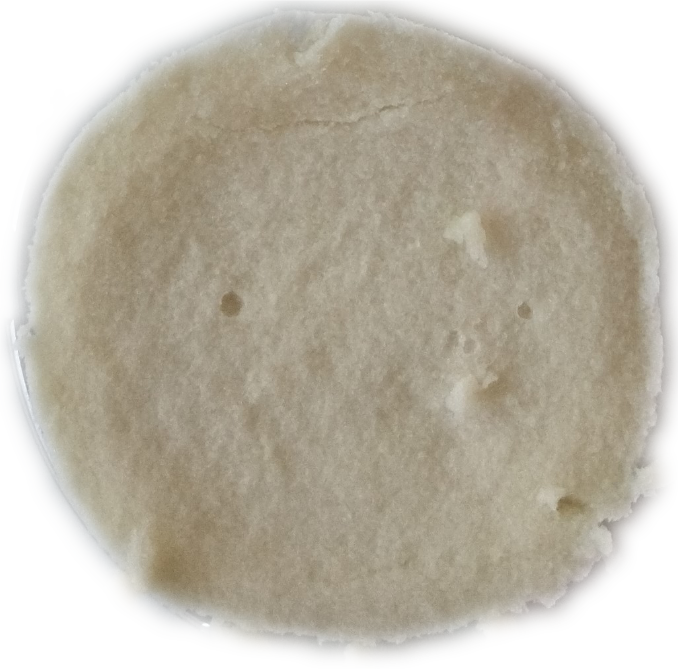 | 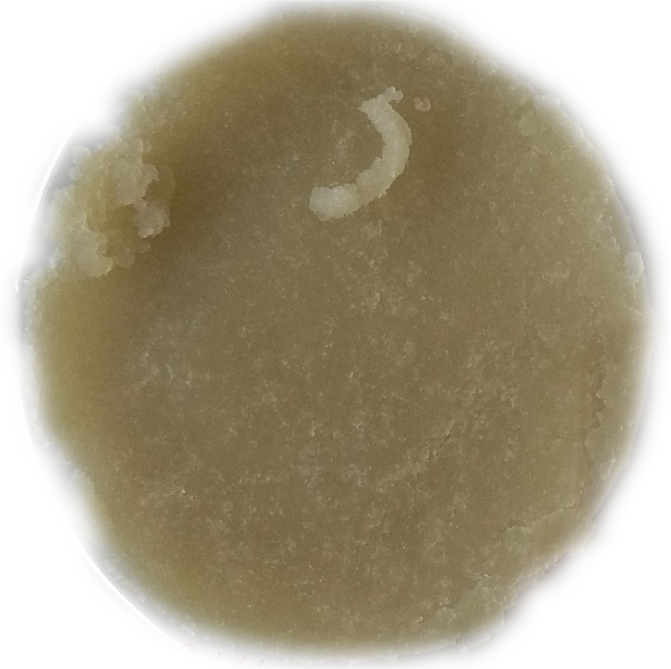 | 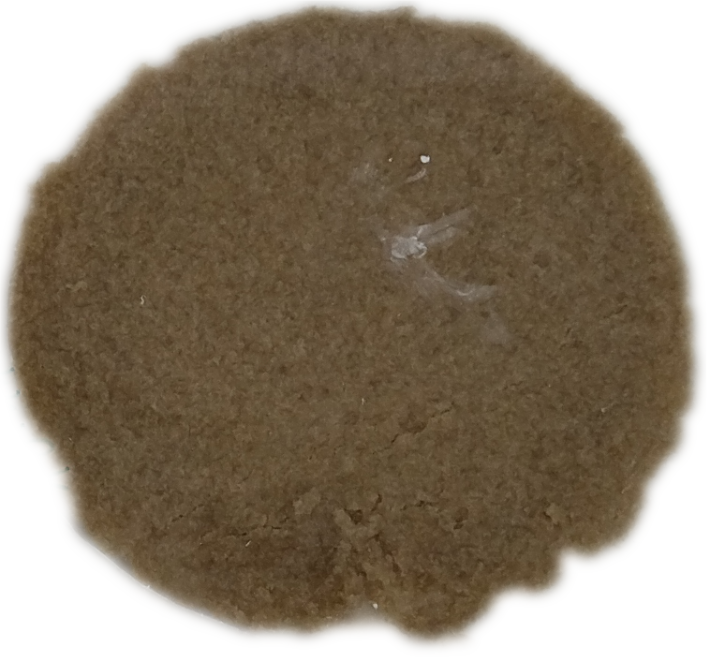 |

**Figure S3 Intercellular liquid content of PCC^Sia^**. Low: complete removal of excess liquid without humidity chamber; medium: partial removal of excess liquid with humidity chamber; High: adding additional drops of water on the filter paper and humidity chamber.

| **A** | 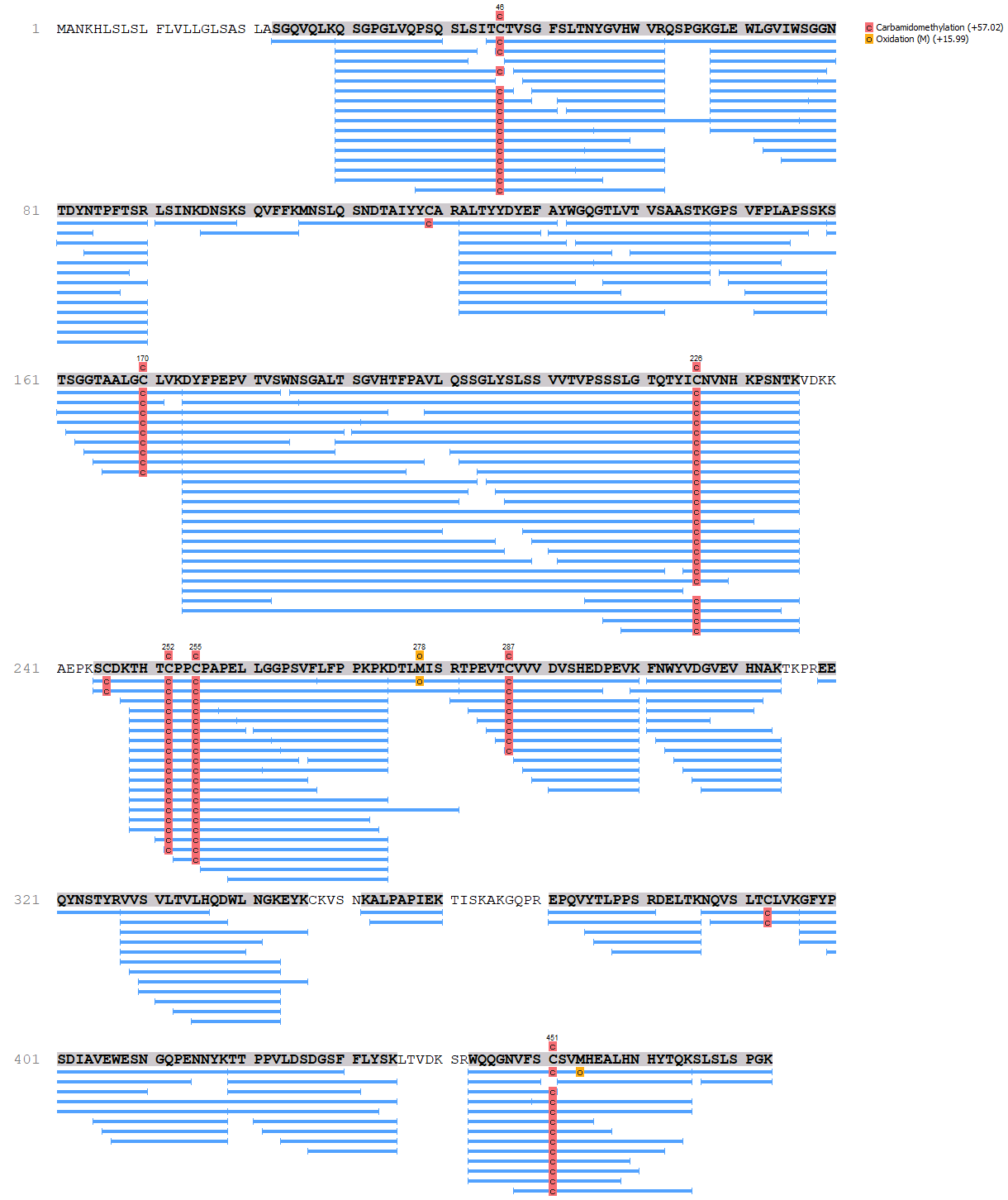 |
| --- | --- |

| **B** | 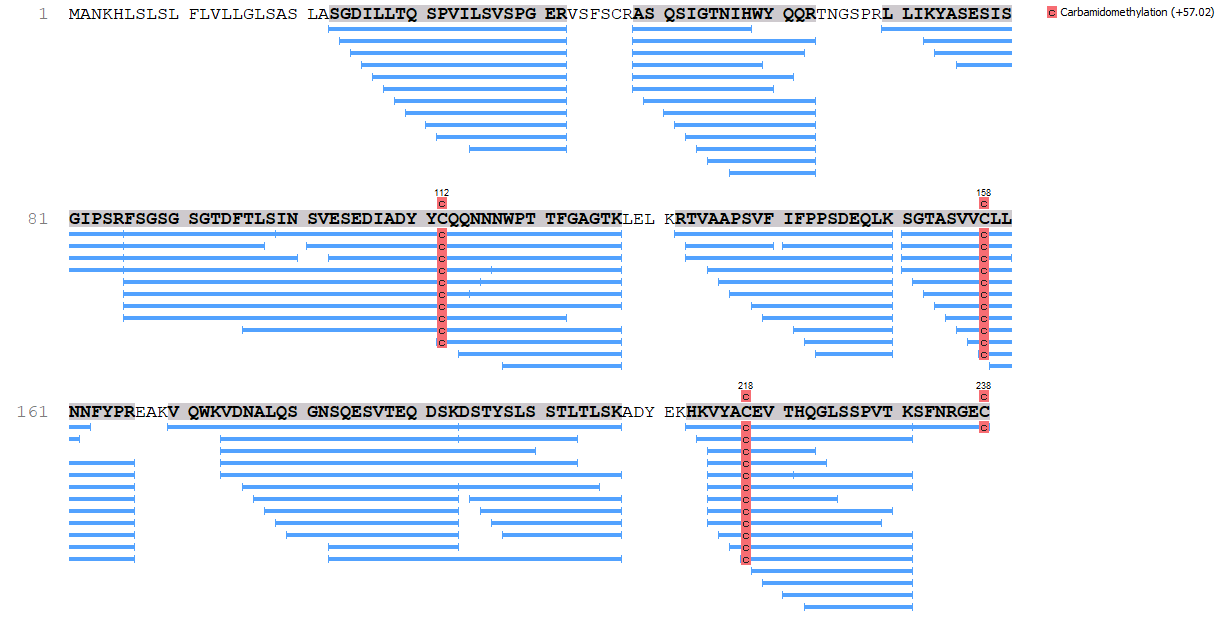 |
| --- | --- |

**Figure S4 Peptide mass-fingerprint analysis and sequence coverage of** **PCC^Sia^ derived cetuximab heavy chain (A) and light chain (B).** Sequence covered is highlighted in gray boxes in bold and the observed individual peptides are represented as blue lines underlying their respective matching sequence; carbamidomethylated cysteines (+57.02 Da) and oxidized methionines (+15.99 Da) are highlighted in red and orange, respectively.


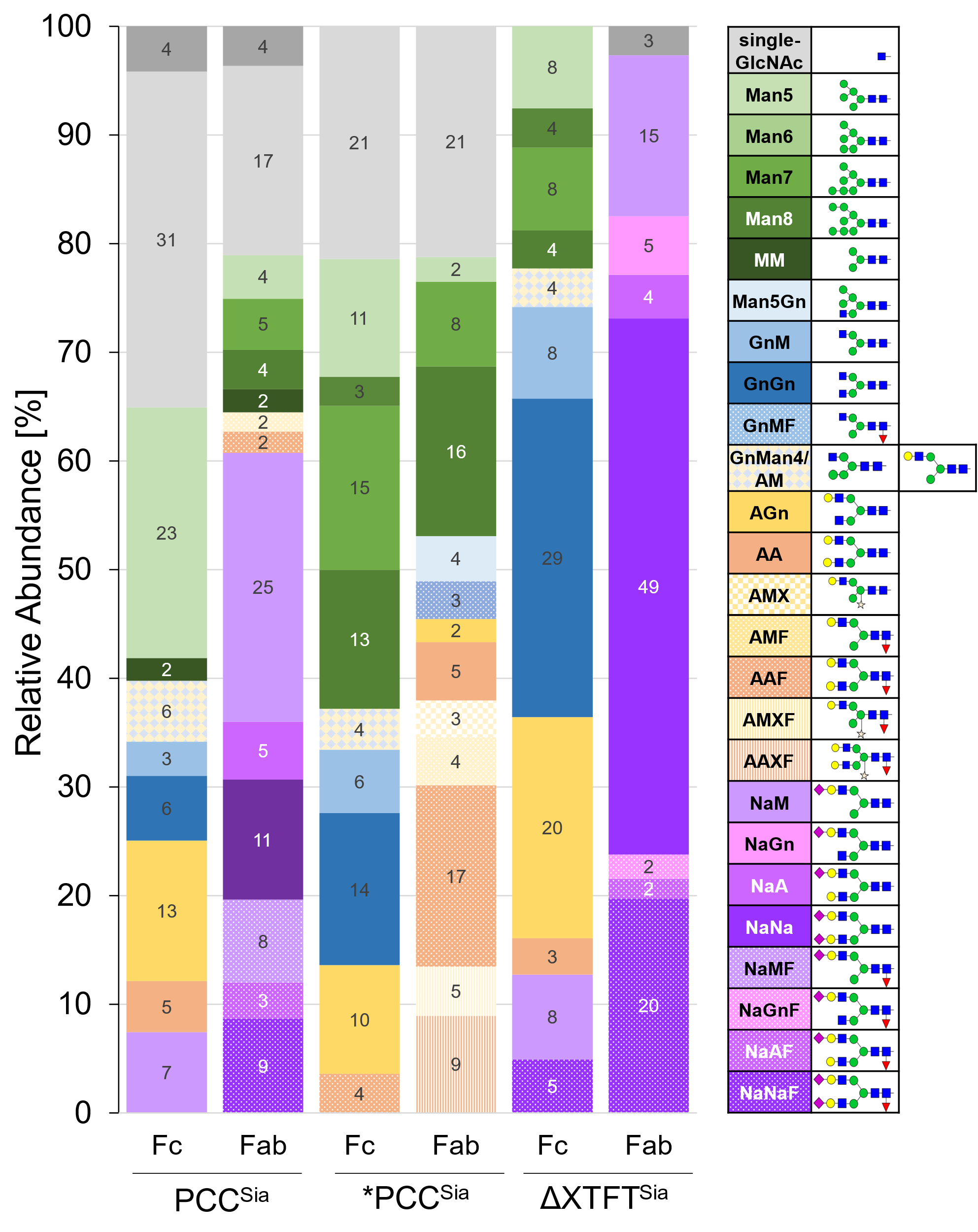


**Figure S5 LC-ESI-MS** based quantified glycoforms from purified cetuximab (Cx) Fc and Fab glycosite. PCC^Sia^ and *PCC^Sia^ is derived from clone 3 and 7, respectively (see SFig 1). Glycan nomenclature according to Altmann et al., (2024).


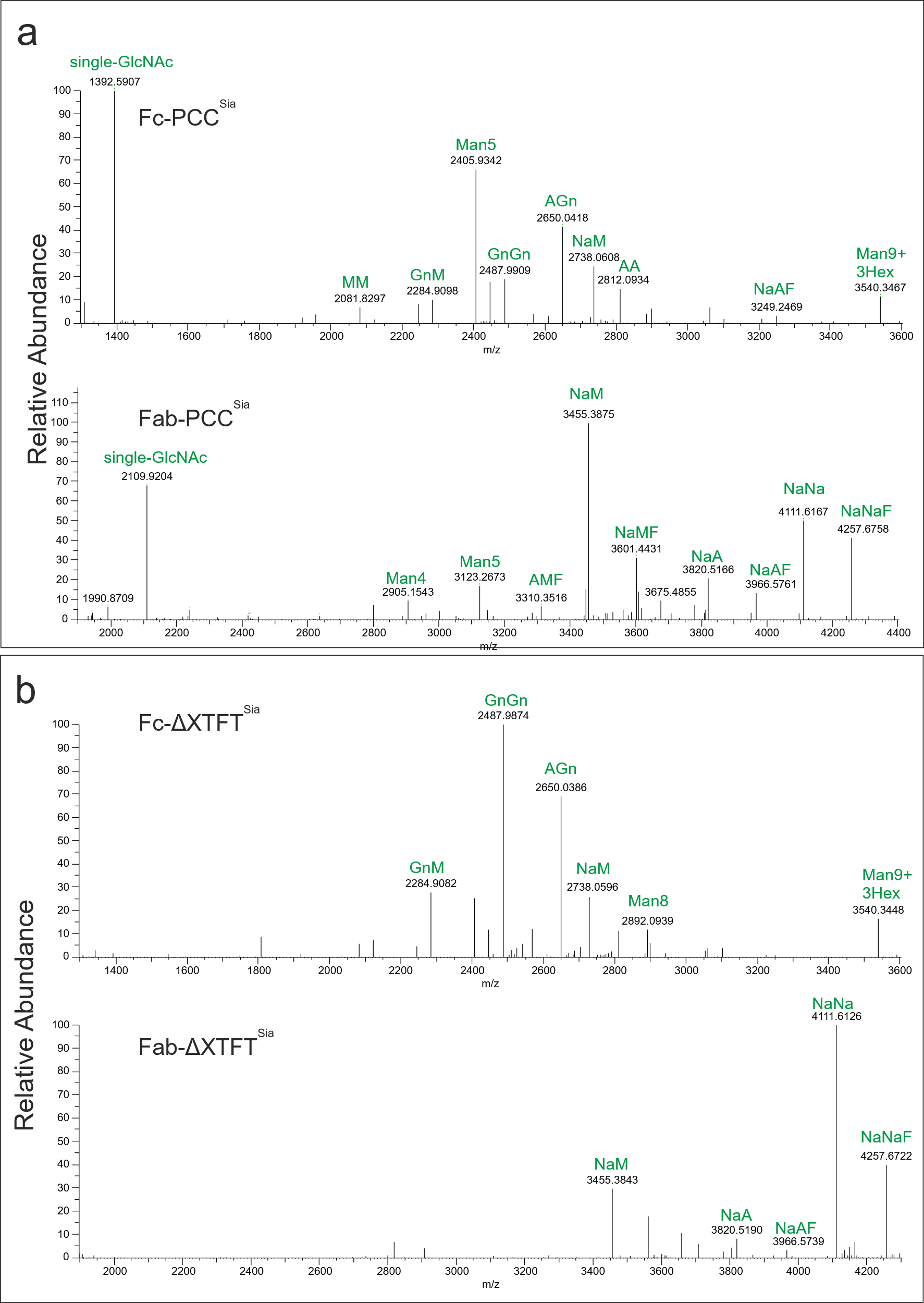


**Figure S6** Examples for LC-ESI-MS spectra of Cx-Fcs and Fab (deconvoluted form) expressed in (a) PCC^Sia^ and in (b) ΔXTFT^Sia^. Fc glycopeptide EEQYNSTYR (1189.5120 Da) with glycosite at N297 and Fab glycopeptide MNSLQSNDTAIYYCAR (1906.8422). Glycan nomenclature according to Altmann et al., (2024).

**Supplementary references**

Altmann, F., Helm, J., Pabst, M., & Stadlmann, J. (2024). Introduction of a human- and keyboard-friendly N-glycan nomenclature. *Beilstein J Org Chem*, *20*, 607-620. https://doi.org/10.3762/bjoc.20.53

Dobersberger, M., Sumesgutner, D., Zajc, C. U., Salzer, B., Laurent, E., Emminger, D., Sylvander, E., Lehner, E., Teufl, M., Seigner, J., Bobbili, M. R., Kunert, R., Lehner, M., & Traxlmayr, M. W. (2024). An engineering strategy to target activated EGFR with CAR T cells. *Cell Rep Methods*, *4*(4), 100728. https://doi.org/10.1016/j.crmeth.2024.100728

Eidenberger, L., Eminger, F., Castilho, A., & Steinkellner, H. (2022). Comparative analysis of plant transient expression vectors for targeted N-glycosylation. *Front Bioeng Biotechnol*, *10*, 1073455. https://doi.org/10.3389/fbioe.2022.1073455

Kallolimath, S., Castilho, A., Strasser, R., Grunwald-Gruber, C., Altmann, F., Strubl, S., Galuska, C. E., Zlatina, K., Galuska, S. P., Werner, S., Thiesler, H., Werneburg, S., Hildebrandt, H., Gerardy-Schahn, R., & Steinkellner, H. (2016). Engineering of complex protein sialylation in plants. *Proc Natl Acad Sci U S A*, *113*(34), 9498-9503. https://doi.org/10.1073/pnas.1604371113

Kogelmann, B., Melnik, S., Bogner, M., Kallolimath, S., Stoger, E., Sun, L., Strasser, R., D'Aoust, M. A., Lavoie, P. O., Saxena, P., Gach, J. S., & Steinkellner, H. (2024). A genome-edited N. benthamiana line for industrial-scale production of recombinant glycoproteins with targeted N-glycosylation. *Biotechnol J*, *19*(1), e2300323. https://doi.org/10.1002/biot.202300323

Patel, D., Lahiji, A., Patel, S., Franklin, M., Jimenez, X., Hicklin, D. J., & Kang, X. (2007). Monoclonal antibody cetuximab binds to and down-regulates constitutively activated epidermal growth factor receptor vIII on the cell surface. *Anticancer Res*, *27*(5A), 3355-3366. https://www.ncbi.nlm.nih.gov/pubmed/17970081

Rademacher, T., Sack, M., Blessing, D., Fischer, R., Holland, T., & Buyel, J. (2019). Plant cell packs: a scalable platform for recombinant protein production and metabolic engineering. *Plant Biotechnol J*, *17*(8), 1560-1566. https://doi.org/10.1111/pbi.13081

Schoberer, J., Konig, J., Veit, C., Vavra, U., Liebminger, E., Botchway, S. W., Altmann, F., Kriechbaumer, V., Hawes, C., & Strasser, R. (2019). A signal motif retains Arabidopsis ER-alpha-mannosidase I in the cis-Golgi and prevents enhanced glycoprotein ERAD. *Nat Commun*, *10*(1), 3701. https://doi.org/10.1038/s41467-019-11686-9

Sun, L., Kallolimath, S., Palt, R., Stiasny, K., Mayrhofer, P., Maresch, D., Eidenberger, L., & Steinkellner, H. (2021). Increased in vitro neutralizing activity of SARS-CoV-2 IgA1 dimers compared to monomers and IgG. *Proc Natl Acad Sci U S A*, *118*(44). https://doi.org/10.1073/pnas.2107148118
